# Development of Simultaneous Drug Concentration Measurement Method Using an Automated Pretreatment Liquid Chromatography/Tandem Mass Spectrometry System for Therapeutic Drug Monitoring

**DOI:** 10.3390/pharmaceutics16091138

**Published:** 2024-08-28

**Authors:** Yu Sato, Hiroki Kondo, Yuji Sato, Ai Abe, Masafumi Kikuchi, Toshihiro Sato, Masaki Kumondai, Kohei Yoshikawa, Yoshihiro Hayakawa, Masamitsu Maekawa, Nariyasu Mano

**Affiliations:** 1 Department of Pharmaceutical Sciences, Tohoku University Hospital, 1-1 Seiryo-machi, Aoba-ku, Sendai 980-8574, Japan; hiroki.kondou.c5@tohoku.ac.jp (H.K.); yuji.sato.a4@tohoku.ac.jp (Y.S.); ai.abe.e8@tohoku.ac.jp (A.A.); masafumi.kikuchi@hos.akita-u.ac.jp (M.K.); toshihiro.sato@tohoku.ac.jp (T.S.); masaki.kumondai.d5@tohoku.ac.jp (M.K.); mano@hosp.tohoku.ac.jp (N.M.); 2 Graduate School of Pharmaceutical Sciences, Tohoku University, 6-3 Aoba, Aramaki, Aoba-ku, Sendai 980-8578, Japan; 3 Shimadzu Corporation, 1 Nishinokyo Kuwabara-cho, Nakagyo-ku, Kyoto 604-8511, Japan; yoshikawa.kohei.4kx@shimadzu.co.jp (K.Y.); yo-haya@shimadzu.co.jp (Y.H.)

**Keywords:** automated sample preparation, therapeutic drug monitoring, LC-MS/MS, plasma concentration, simultaneous measurement method of drug efficacy

## Abstract

Therapeutic drug monitoring (TDM) is a personalized treatment approach that involves optimizing drug dosages based on patient-specific factors, such as drug plasma concentrations, therapeutic efficacy, or adverse reactions. The plasma concentration of drugs is determined using liquid chromatography/tandem mass spectrometry (LC-MS/MS) or various immunoassays. Compared with immunoassays, LC-MS/MS requires more pretreatment time as the number of samples increases. Recently, fully automated pretreatment LC-MS/MS systems have been developed to automatically perform whole-sample pretreatment for LC-MS/MS analysis. In this study, we developed a method for simultaneous concentration determination of five analytes (clozapine, mycophenolic acid, sunitinib, *N*-desethylsunitinib, and voriconazole) using LC-MS/MS for clinical TDM using a fully automated LC-MS/MS pretreatment system. In the developed method, the intra- and inter-assay relative error (RE) values ranged between −14.8% and 11.3%; the intra- and inter-assay coefficient of variation (CV) values were <8.8% and <10.5%, respectively. The analytes showed good stability, with RE values ranging between −13.6% and 10.9% and CV values <8.9%. Furthermore, the plasma concentrations in clinical samples using this method and the conventional manual pretreatment method showed similar results. Therefore, the method developed in this study could be considered a useful pretreatment method for routine TDM in patients.

## 1. Introduction

Therapeutic drug monitoring (TDM) is a personalized treatment approach that involves optimizing drug dosages based on patient-specific factors, such as drug plasma concentrations, therapeutic efficacy, or adverse reactions [1,2]. TDM is an effective strategy for adjusting dosages based on an effective therapeutic range, confirming medication adherence, and evaluating drug tolerance and interactions [3]. For instance, sunitinib, a drug used to treat renal cell carcinoma, requires maintenance of a combined plasma trough concentration of its active metabolite, *N*-desethylsunitinib (SU-12662), above 50 ng/mL for optimal antitumor effects [3,4]. However, concentrations exceeding 100 ng/mL increase the risk of dose-limiting toxicity [5]. Mycophenolic acid, an immunosuppressant used for solid organ transplantation, has a narrow therapeutic range and high interindividual variability [6]. The antipsychotic clozapine exhibits a link between blood concentration and both efficacy and adverse reactions, such as sleepiness and seizures [7,8]. This necessitates monitoring for plasma concentrations and dosage adjustments based on individual patient conditions and treatment targets. Therefore, TDM for clozapine is recommended to achieve optimal therapeutic efficacy while minimizing side effects [9,10]. Based on these considerations, TDM for these drugs has become a routine clinical practice in Japan.

The various methods employed for TDM to measure blood drug concentrations are categorized into two groups: immunological methods, such as enzyme-linked immunosorbent assays, and separation analysis methods, such as gas chromatography, liquid chromatography with ultraviolet detection (LC-UV), and liquid chromatography/tandem mass spectrometry (LC-MS/MS). Immunological methods depend on drug-specific binding to antibodies [11,12]. These methods allow quick and simple quantification without the need for specialized techniques. However, new drugs or drugs with low measurement frequencies may not have commercially available antibodies. Moreover, there is a risk of overestimating the quantification value due to cross-reactions with less specific antigens. In contrast, separation analysis methods, such as LC-UV and LC-MS/MS, offer high accuracy and versatility for the simultaneous quantification of multiple components [13,14,15,16]. However, these methods also have limitations, such as time-consuming analysis, manual sample preparation, and the need for user training [15]. As the number of samples increases, the preprocessing time also increases, requiring automation. Recently, the Shimadzu Corporation has developed a fully automatic pretreatment LC-MS/MS system that can automatically perform all processes from sample preparation to LC-MS/MS analysis after the user simply inserts the collected plasma into the system following centrifugation of the blood specimen. This system significantly reduces pretreatment time from the 15 min required for conventional manual processing to just 3 min. Additionally, this system was used to measure the levels of immunosuppressive drugs [17,18], beta-lactam antibiotics [19], organic acids [20], uracil [21], steroids [22], and modified nucleosides [23].

We previously reported on methods for measuring blood concentrations among various patient groups: mycophenolic acid and voriconazole in patients undergoing lung transplantation [14], sunitinib and *N*-desethylsunitinib in patients with renal cell carcinoma [24], and clozapine in patients with psychiatric disorders [25]. However, adapting to the optimal method for each individual measurement is a time-consuming process. In this study, we developed a universal method for simultaneously quantifying five analytes (clozapine, mycophenolic acid, sunitinib, *N*-desethylsunitinib, and voriconazole) using LC-MS/MS in clinical TDM to improve performance in routine clinical practice. This method utilizes CLAM-2030 (Shimadzu Corporation, Kyoto, Japan), which is a fully automated LC-MS/MS preprocessing system. Furthermore, we assessed clinical specimens using both the conventional manual pretreatment method and our newly developed method. The comparison between results demonstrated the efficacy and practicality of our new approach for routine clinical use.

## 2. Materials and Methods

### 2.1. Chemicals and Reagents

Clozapine, sunitinib, mycophenolic acid, mycophenolic acid-d3, voriconazole, and voriconazole-d3 were purchased from Toronto Research Chemicals (Toronto, ON, Canada). Clozapine-d8 was purchased from R&D Systems (Minneapolis, MN, USA). *N*-Desethylsunitinib was purchased from MedChemExpress (Monmouth, NJ, USA). Sunitinib-d10 was purchased from Alsachim (Illkirch-Graffenstaden, France). High-performance LC-grade ammonium formate, formic acid, and methanol were purchased from FUJIFILM WAKO Pure Chemical Corporation (Osaka, Japan). Ultrapure water was prepared using a Puric-α apparatus and used for LC-MS/MS analysis. All other chemicals used had the highest commercially available purity.

### 2.2. Preparation of Stock Solutions, Working Solutions, Calibration Standard Samples, and Quality Control Samples

**Figure 1:**
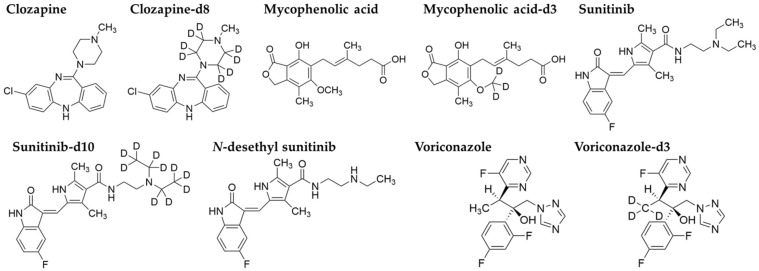
Chemical structures of clozapine, clozapine-d8, mycophenolic acid, mycophenolic acid-d3, sunitinib, sunitinib-d10, *N*-desethylsunitinib, voriconazole, and voriconazole-d3.

The chemical structures are presented in Figure 1. Stock solutions of clozapine (200 µg/mL), sunitinib (10 µg/mL), sunitinib-d10 (1 mg/mL), *N*-desethylsunitinib (10 µg/mL), mycophenolic acid (2 mg/mL), mycophenolic acid-d3 (1 mg/mL), voriconazole (2 mg/mL), voriconazole-d3 (1 mg/mL) in methanol, and clozapine-d8 (100 μg/mL) in ethanol were prepared and stored at −20 °C. Stock solutions were diluted with human plasma to prepare calibration standard (CS) and quality control (QC) samples, which were stored in light-resistant polypropylene tubes at 4 °C. Details of the CS and QC samples are presented in Table 1. The internal standard (IS) mix solution comprised clozapine-d8 (100 µg/mL), sunitinib-d10 (1 mg/mL), mycophenolic acid-d3 (1 mg/mL), and voriconazole-d3 (1 mg/mL), each diluted and mixed with methanol to 1 µg/mL.

### 2.3. Sample Preparation

#### 2.3.1. Automated Method (CLAM Method)

**Figure 2:**
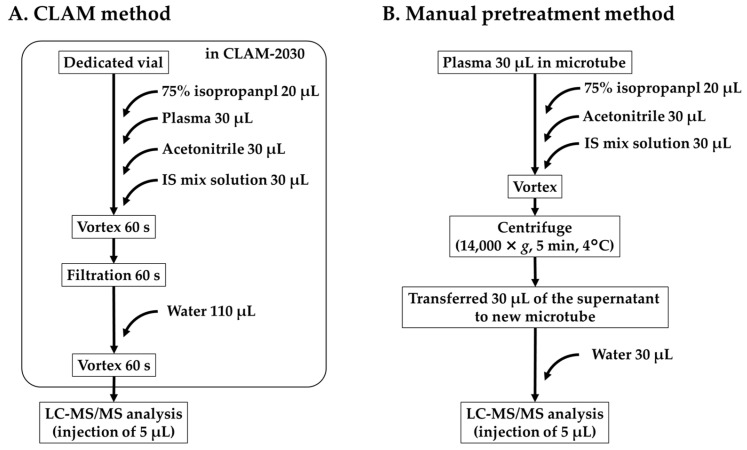
Sample pretreatment process for the CLAM method (**A**) and manual pretreatment methods (**B**). Squares represent the steps automatically processed using CLAM-2030.

For this study, CLAM-2030 (Shimadzu, Kyoto, Japan) was used as the automated system (CLAM method). Regarding the CLAM method, a dedicated filter (polytetrafluoroethylene membrane with 0.45 µm pores) and vials were used for pretreatment. First, 20 µL of isopropanol (75%) was dispensed onto the dedicated filter to activate its hydrophobic properties. Next, the plasma sample, IS mix solution, and acetonitrile (30 µL each) were dispensed into a dedicated vial and stirred for 60 s. The samples were filtered using a vacuum (approximately 50–60 kPa) for 60 s in the filtration unit. The filtered samples were diluted with 110 µL of water and stirred for 10 s; 5 µL of the mixture was injected into the LC-MS/MS system (Figure 2A).

#### 2.3.2. Manual Pretreatment Method

Thirty microliters of each sample was diluted with an equal volume of acetonitrile in a 1.5 mL microtube, and 30 µL of IS mix solution was added. The mixture was vortexed and centrifuged at 14,000× *g* for 5 min at 4 °C. After centrifugation, 30 μL of the supernatant was transferred to a new 1.5 mL microtube and diluted with water (30 μL). Five microliters of the mixture were injected into the LC-MS/MS system (Figure 2B).

### 2.4. LC-MS/MS Conditions

LC-MS/MS analysis was performed using an LCMS-8050 triple quadrupole mass spectrometer coupled to a Nexera X2 ultra-high-performance LC system (Shimadzu, Kyoto, Japan). Data acquisition and processing were performed using OpenSolution Quant Analytics (Shimadzu). The Nexera X2 system comprised a vacuum degasser, two solvent delivery systems, an autosampler, and a column oven. Chromatographic separation was achieved using a YMC-Triart C18 column (50 mm × 2.1 mm, S-3 µm, 12 nm; YMC, Kyoto, Japan). The column temperature was maintained at 40 °C. The flow rate was set at 0.3 mL/min; the injection volume was 5 μL for analysis. Mobile phases A and B comprised 20 mM aqueous ammonium formate adjusted to pH 3.6 (A) and methanol (B), respectively. The gradient program was as follows: 0–0.25 min, 40% B; 0.25–2.5 min, 40–100% B; 2.5–3 min, 100% B; and 3.01–4 min, 40% B. The LCMS-8050 was equipped with an electrospray ionization source operating in the positive ion detection mode. The conditions for MS analysis were as follows: interface voltage, 4.0 kV; desolvation line temperature, 250 °C; block heater temperature, 400 °C; interface temperature, 300 °C; nebulizing gas flow, 3 L/min; drying gas flow, 10 L/min; and heating gas flow, 10 L/min.

### 2.5. Validation

The analytical method was validated in accordance with the US Food and Drug Administration (FDA) guidelines for the validation of bioanalytical assays [26] and the European Medicines Agency (EMA) guidelines for bioanalytical method validation [27].

Intra-day precision and accuracy were assessed by analyzing five replicate QC samples at four different levels [lower limit of quantification (LLOQ), low QC (LQC), medium QC (MQC), and high QC (HQC)] on the same day. Inter-day precision and accuracy were estimated by analyzing the QC samples in three analytical runs. The stability of each analyte in human plasma was investigated using LOQ, MOQ, and HQC samples at room temperature for 24 h, 4 °C for 24 h, and 4 °C for 28 days.

### 2.6. Measurement of Patient Samples

This study was approved by the Ethical Review Committee of Tohoku University Graduate School of Medicine (approval number: 2023-1-291). Patient consent was obtained by implementing an opt-out method for patient samples measured by routine TDM. Patient blood samples were collected using EDTA collection tubes and centrifuged at 1580× *g* for 10 min. Subsequently, the plasma samples were collected and stored at 4 °C until analysis.

### 2.7. Data Analysis

Concordance between the CLAM and conventional manual pretreatment methods was assessed by conducting Passing–Bablok and Bland–Altman analyses [28,29]. Spearman rank correlation, Bland–Altman, and Passing–Bablok analyses were performed using MedCalc statistical software version 22 (MedCalc, Ostend, Belgium). Statistical significance was set at *p* < 0.05.

## 3. Results and Discussion

### 3.1. Development and Validation of the CLAM Method

First, we modified our previous study [14,24,25] and determined the MS conditions that could be used to detect the five analytes (clozapine, mycophenolic acid, sunitinib, N-desethylsunitinib, and voriconazole). The MS parameters are listed in Table 2. Considering voriconazole, saturation of the detection signal was observed therefore, an in-source collision-induced dissociation technique [14,25,30,31], which could reduce the number of ions introduced into the mass spectrometer, was used for detection.

**Figure 3:**
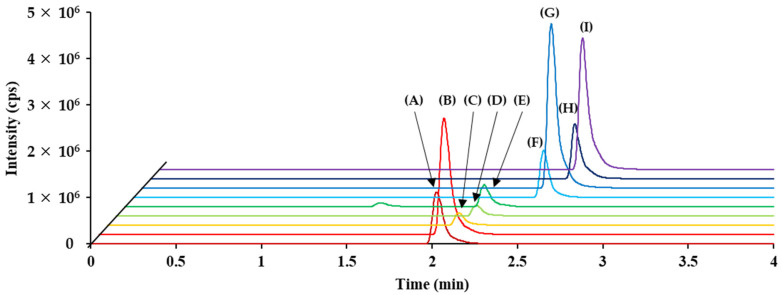
LC-MS/MS chromatograms of the MQC sample for (**A**) clozapine, (**B**) clozapine-d8, (**C**) *N*-desethylsunitinib, (**D**) sunitinib, (**E**) sunitinib-d10, (**F**) voriconazole, (**G**) voriconazole-d3, (**H**) mycophenolic acid, and (**I**) mycophenolic acid-d3.

**Figure 4:**
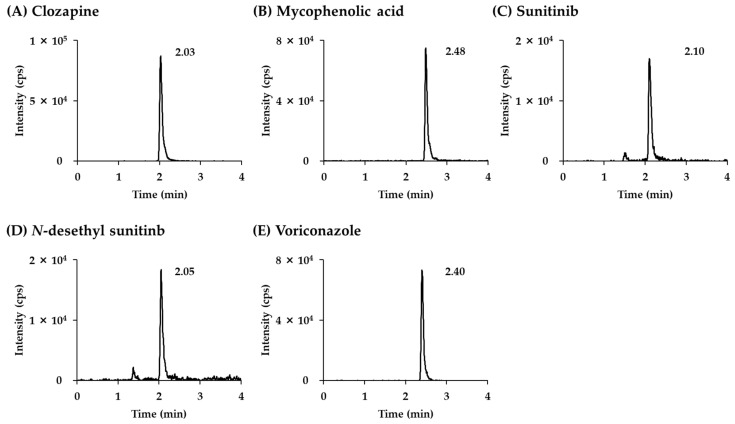
LC-MS/MS chromatograms of LLOQ sample for (**A**) clozapine, (**B**) mycophenolic acid, (**C**) sunitinib, (**D**) *N*-desethylsunitinib, and (**E**) voriconazole.

The retention time for each analyte was less than 3 min, as illustrated in the LC-MS/MS chromatogram shown in Figure 3 (five analytes and four ISs). Additionally, Figure 4 presents the LC-MS/MS chromatograms for the LLOQ level of each individual analyte. Linearity was observed in the five analytes (clozapine, mycophenolic acid, sunitinib, *N*-desethylsunitinib, and voriconazole) using the CLAM method over the working range listed in Table 1.

The calibration regression curves (*y*) and correlation coefficients (*R*^2^) are presented in Table 3. The entire concentration range was linear (*R*^2^ >0.99), confirming the feasibility of quantitative analysis using the CLAM method. The accuracy and precision of the CLAM method were assessed at three QC levels and the LLOQ (Table 4). The intra-day relative error (RE) values were between −14.8% and 11.3%, and the coefficient of variation (CV) values were all less than 8.8%. The inter-day RE values were between −5.4% and 11.2%, and the CV values were all less than 10.5%. These results indicated that the CLAM method could measure each analyte with the same accuracy and reproducibility as our previously reported methods [14,24,25]. The stability data are presented in Table 5. Across all stability tests, the analytes exhibited high stability, with RE values between −13.6% and 10.9% and CV values below 8.9%. Long-term storage of up to 28 days in the refrigerator did not affect the assay results, suggesting that transportation of samples from the collection facility to the analysis facility while keeping them refrigerated is feasible. We consider this finding valuable for the practical application of the CLAM method to routine TDM.

### 3.2. Method Comparison

**Figure 5:**
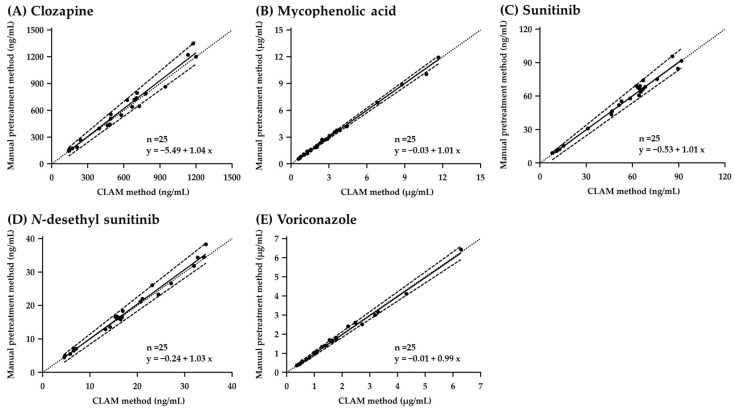
Passing–Bablok regression analysis of the CLAM and manual pretreatment methods for (**A**) clozapine, (**B**) mycophenolic acid, (**C**) sunitinib, (**D**) *N*-desethylsunitinib, and (**E**) voriconazole. The thick solid line indicates the estimated regression equation, the dashed lines indicate the upper and lower limits of the 95% confidence interval, and the dotted line indicates the identity line (*x* = *y*).

**Figure 6:**
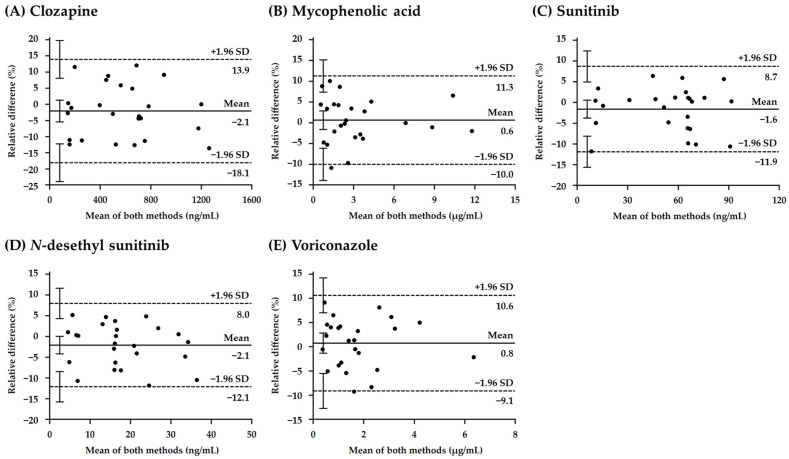
Bland–Altman plots for (**A**) clozapine, (**B**) mycophenolic acid, (**C**) sunitinib, (**D**) *N*-desethylsunitinib, and (**E**) voriconazole. The relative difference is calculated using the following equation: (CLAM method − manual pretreatment method)/mean of both methods × 100. The solid line indicates the mean relative difference between the two assays, whereas the dashed line indicates the upper and lower limits of agreement calculated as the mean relative difference ± 1.96 standard deviations.

Comparison of the plasma concentrations of the five analytes (clozapine, mycophenolic acid, sunitinib, *N*-desethylsunitinib, and voriconazole) measured using the CLAM and conventional manual pretreatment methods was conducted using Passing–Bablok regression (Figure 5) [28] and Bland–Altman (Figure 6) [29] analyses.

The slopes and intercepts of the Passing–Bablok regression for each analyte are listed in Table 6. Regarding all analytes, the slope of the 95% confidence interval (CI) was across one, indicating no proportional bias, and the intercept of the 95% CI was across zero, indicating no additional constant bias. The Spearman’s rank correlation coefficients between the CLAM and manual pretreatment methods were 0.978, 0.997, 0.937, 0.969, and 0.997 for clozapine, MPA, sunitinib, *N*-desethylsunitinib, and voriconazole, respectively, indicating a high correlation for all analytes (Table 6). Bland–Altman analysis showed a mean bias of −2.08% (95% CI, −5.45 to 1.29), 0.63% (95% CI, −1.61 to 2.87), –1.59% (95% CI, −3.75 to 0.58), –2.07% (95% CI, −4.18 to 0.04), and 0.77% (95% CI, −1.31 to 2.84) for clozapine, MPA, sunitinib, *N*-desethylsunitinib, and voriconazole, respectively. The 95% CI for all analytes was across zero, indicating no systematic error. Additionally, the upper [mean bias +1.96 standard deviations (SD)] and lower (mean bias −1.96 SD) limits of agreement were calculated for each compound (Figure 5). These results suggest that there is no significant bias between the CLAM and manual pretreatment methods and that there is a strong correlation between the methods. Moreover, the proportional bias of the five analyte concentrations between the CLAM and conventional manual pretreatment methods was within 20% for all samples, meeting the criteria for sample reanalysis (US FDA guidelines for the validation of bioanalytical assays and EMA guidelines for bioanalytical method validation) [26,27]. Therefore, using the CLAM method, the results were comparable to those of the conventional manual pretreatment method in terms of assay outcomes. These results suggest that automation of conventional pretreatment method processes such as reagent addition, mixing, deproteinization, and centrifugation could reduce the required skills of the user to a minimum and contribute to the reduction in routine TDM. However, in this study, although we compared the automated pretreatment method using the CLAM method with manual pretreatment, we did not evaluate it against other automated pretreatment systems, which might be a limitation of this study. Therefore, future studies comparing this method with other automated pretreatment systems may provide a more comprehensive demonstration of its clinical value.

## 4. Conclusions

We established a simultaneous quantitative method for five drugs (clozapine, mycophenolic acid, sunitinib, *N*-desethylsunitinib, and voriconazole) using LC-MS/MS in clinical TDM with CLAM-2030 (Shimadzu Corporation), a fully automated LC-MS/MS preprocessing system. The validated CLAM method led to results comparable to those obtained using conventional manual pretreatment methods. Therefore, the CLAM method might be considered a valuable pretreatment method for routine TDM, offering both efficiency and reliability in clinical settings.

## Figures and Tables

**Table 1:** Concentration of the five analytes in the calibration standard and quality control samples.

Sample	Clozapine (ng/mL)	Mycophenolic Acid (µg/mL)	Sunitinib (ng/mL)	*N*-Desethylsunitinib (ng/mL)	Voriconazole (µg/mL)
CS1	30	0.2	1	1	0.1
CS2	60	0.4	2	2	0.2
CS3	150	1	5	5	0.5
CS4	750	5	25	25	2.5
CS5	1500	10	50	50	5
CS6	3000	20	100	100	10
LLOQ	30	0.2	1	1	0.1
LQC	100	0.66	3.3	3.3	0.33
MQC	300	2	10	10	1
HQC	1000	6.6	33	33	3.3

CS, calibration standard; LLOQ, lower limits of quantification; LQC, low quality control; MQC, medium quality control; HQC, high quality control.

**Table 2:** Optimized selected reaction monitoring (SRM) settings for each analyte.

Compound	Type	SRM Transition (*m/z*)	Q1 (V)	CE (V)	Q3 (V)	Q-Array Bias (V)	RT (min)	IS
Clozapine	Analyte	328.70 > 272.10	−23.0	−23.0	−19.0	-	2.03	Clozapine-d8
Clozapine-d8	IS	336.90 > 276.10	−24.0	−24.0	−30.0	-	2.02	-
Mycophenolic acid	Analyte	321.40 > 207.00	−11.0	−20.0	−22.0	-	2.48	Mycophenolic acid-d3
Mycophenolic acid-d3	IS	324.05 > 210.05	−16.0	−22.0	−22.0	-	2.48	-
Sunitinib	Analyte	399.10 > 283.05	−20.0	−28.0	−20.0	-	2.10	Sunitinib-d10
*N*-desethylsunitinib	Analyte	371.10 > 283.10	−19.0	−22.0	−20.0	-	2.05	Sunitinib-d10
Sunitinib-d10	IS	409.50 > 283.55	−13.0	−24.0	−10.0	-	2.11	-
Voriconazole	Analyte	349.85 > 281.10	−13.0	−16.0	−13.0	100	2.40	Voriconazole-d3
Voriconazole-d3	IS	352.90 > 284.10	−13.0	−17.0	−20.0	-	2.40	-

CE, collision energy; RT, retention time; IS, internal standard.

**Table 3:** Linearity of the five analytes using the CLAM method.

Analyte	Range	Calibration Regression Curves	*R* ^2^
Clozapine	30–3000 ng/mL	*y* = 0.0017x − 0.0017	0.9986
Mycophenolic acid	0.2–20 µg/mL	*y* = 0.1741x − 0.0056	0.9993
Sunitinib	1–100 ng/mL	*y* = 0.0668x + 0.0175	0.9989
*N*-desethylsunitinib	1–100 ng/mL	*y* = 0.0833x + 0.0337	0.9976
Voriconazole	0.1–10 µg/mL	*y* = 0.2430x + 0.0046	0.9978

**Table 4:** Assay performance of the five analytes in four quality control samples.

Analyte	Intra-Day (*n* = 5)		Inter-Day (*n* = 15)	
	RE (%)	CV (%)	RE (%)	CV (%)
Clozapine				
LLOQ	6.2	2.8	7.4	5.7
LQC	−1.6	1.2	−0.4	3.0
MQC	0.1	1.1	−3.0	4.2
HQC	1.2	0.7	1.3	3.7
Mycophenolic acid				
LLOQ	11.3	5.1	11.2	3.8
LQC	−2.8	2.7	1.6	4.6
MQC	−5.1	2.2	−2.7	3.8
HQC	−5.7	2.8	0.7	5.9
Sunitinib				
LLOQ	−10.7	5.8	−1.2	9.9
LQC	−4.1	4.2	0.9	7.2
MQC	4.3	2.2	0.8	6.7
HQC	−0.4	0.8	−1.5	8.2
*N*-desethylsunitinib				
LLOQ	−14.8	8.8	−4.2	10.5
LQC	−2.9	2.2	−0.5	6.2
MQC	3.8	2.3	3.0	5.2
HQC	−0.1	1.4	0.8	5.7
Voriconazole				
LLOQ	−2.6	3.1	−5.4	8.2
LQC	2.4	1.2	4.6	3.8
MQC	4.6	1.9	5.9	5.1
HQC	3.5	1.6	7.4	4.8

RE, relative error; CV, coefficient of variation.

**Table 5:** Stability of the five analytes in three quality control samples (*n* = 5).

Condition	Clozapine		Mycophenolic Acid		Sunitinib		*N*-Desethylsunitinib		Voriconazole	
	RE (%)	CV (%)	RE (%)	CV (%)	RE (%)	CV (%)	RE (%)	CV (%)	RE (%)	CV (%)
Room temperature for 24 h										
LQC	−13.1	6.2	−0.8	2.8	5.9	4.5	8.1	3.3	−5.4	3.6
MQC	−5.6	3.2	6.4	6.7	7.2	5.4	10.9	4.5	8.5	5.2
HQC	−11.0	6.0	−6.8	4.3	1.1	6.4	5.5	3.7	−3.4	2.8
4 °C for 24 h										
LQC	−3.1	1.6	0.3	1.9	1.2	3.2	−1.2	7.4	1.6	0.9
MQC	−8.4	4.0	−5.4	2.5	−3.5	6.1	5.4	5.5	0.5	0.9
HQC	−3.1	1.8	−0.7	0.8	−1.8	7.4	3.4	7.2	4.5	1.9
4 °C for 28 days										
LQC	−7.0	5.5	−6.7	5.0	−3.3	4.1	1.4	1.9	−3.0	5.5
MQC	−12.3	6.5	−10.6	5.8	−9.6	5.1	−0.2	2.2	−4.3	3.9
HQC	−9.4	5.8	−7.6	6.2	−1.8	3.7	4.2	4.1	−2.2	3.9

RE, relative error; CV, coefficient of variation.

**Table 6:** Comparison of the CLAM and manual pretreatment methods for the five analytes determined by Passing–Bablok regression analysis and Spearman’s correlation.

Analyte	Intercept (95% CI)	Slope (95% CI)	Significance (CUSUM Test for Linearity)	Correlation (*r*)	Significance (Spearman’s Correlation)
Clozapine	−5.49 (−49.68–23.59)	1.04 (0.97–1.13)	0.82	0.978	<0.0001
Mycophenolic acid	−0.03 (−0.08–0.06)	1.01 (0.97–1.03)	0.99	0.997	<0.0001
Sunitinib	−0.53 (−4.90–0.68)	1.01 (0.98–1.11)	0.82	0.937	<0.0001
*N*-desethylsunitinib	−0.24 (−1.45–0.35)	1.03 (0.99–1.11)	0.99	0.969	<0.0001
Voriconazole	−0.01 (−0.06–0.05)	0.99 (0.95–1.04)	0.82	0.995	<0.0001

CI, confidence interval.

## Data Availability

The dataset is available on request from the authors.
